# Mucin1 induced trophoblast dysfunction in gestational diabetes mellitus via Wnt/β-catenin pathway

**DOI:** 10.1186/s40659-023-00460-3

**Published:** 2023-08-22

**Authors:** Shuang-Shuang Cui, Ping Zhang, Lu Sun, Yu-Lin-Lan Yuan, Jingyun Wang, Feng-Xiang Zhang, Ruiman Li

**Affiliations:** 1 Department of Gynecology and Obstetrics, The First Affiliated Hospital of Jinan University, Jinan University, Guangzhou, 510632 China; 2 Division of Histology and Embryology, Key Laboratory for Regenerative Medicine of the Ministry of Education, Jinan University, Guangzhou, 510632 China; 3 Fifth Department of Medicine (Nephrology/Endocrinology/Rheumatology/Pneumology), University Medical Centre Mannheim, University of Heidelberg, Mannheim, Germany; 4 State Key Laboratory for Chemistry and Molecular Engineering of Medicinal Resources, Collaborative Innovation Center for Guangxi Ethnic Medicine, School of Chemistry and Pharmaceutical Sciences, Guangxi Normal University, Guilin, 541004 China

**Keywords:** Mucin1, Wnt pathway, Apoptosis, Glucose uptake, Trophoblast, Gestational diabetes mellitus

## Abstract

**Background:**

To elucidate the role of Mucin1 (MUC1) in the trophoblast function (glucose uptake and apoptosis) of gestational diabetes mellitus (GDM) women through the Wnt/β-catenin pathway.

**Methods:**

Glucose uptake was analyzed by plasma GLUT1 and GLUT4 levels with ELISA and measured by the expression of GLUT4 and INSR with immunofluorescence and Western blotting. Apoptosis was measured by the expression of Bcl-2 and Caspase3 by Western blotting and flow cytometry. Wnt/β-catenin signaling measured by Western blotting. In vitro studies were performed using HTR-8/SVneo cells that were cultured and treated with high glucose (HG), sh-MUC1 and FH535 (inhibitor of Wnt/β-catenin signaling).

**Results:**

MUC1 was highly expressed in the placental trophoblasts of GDM, and the Wnt/β-catenin pathway was activated, along with dysfunction of glucose uptake and apoptosis. MUC1 knockdown resulted in increased invasiveness and decreased apoptosis in trophoblast cells. The initial linkage between MUC1, the Wnt/β-catenin pathway, and glucose uptake was confirmed by using an HG-exposed HTR-8/SVneo cell model with MUC1 knockdown. MUC1 knockdown inhibited the Wnt/β-catenin signaling pathway and reversed glucose uptake dysfunction and apoptosis in HG-induced HTR-8/SVneo cells. Meanwhile, inhibition of Wnt/β-catenin signaling could also reverse the dysfunction of glucose uptake and apoptosis.

**Conclusions:**

In summary, the increased level of MUC1 in GDM could abnormally activate the Wnt/β-catenin signaling pathway, leading to trophoblast dysfunction, which may impair glucose uptake and induce apoptosis in placental tissues of GDM women.

**Supplementary Information:**

The online version contains supplementary material available at 10.1186/s40659-023-00460-3.

## Background

Gestational diabetes mellitus (GDM), defined as abnormal glucose metabolism and insulin resistance during pregnancy, has been increasingly prevalent and estimated to affect one in every six pregnancies worldwide in recent decades [1]. The occurrence of GDM increases the occurrence of gestational complications and adverse fetal outcomes [2–4]. Women with GDM tend to develop long-term complications, including type 2 diabetes mellitus (T2DM), hypertension, vascular dysfunction, and nonalcoholic fatty liver disease, among others [5, 6]. Moreover, offspring also have a high risk for the occurrence of T2DM or obesity [7]. These potential adverse outcomes in both mother and offspring underpin the significance of investigating the pathogenesis of GDM.

In GDM-complicated pregnancies, placentas exhibit dysfunction of trophoblast cells compared to normal pregnancies [8, 9]. The hyperglycemic intrauterine environment affects not only the fetus but also placental development and function in GDM. The underlying mechanisms of GDM remain unclear, but some evidence indicates alterations in trophoblast differentiation, invasion, proliferation, apoptosis and the cell cycle, which are required for placental development [10, 11]. Recent studies have already revealed the involvement of genetic and epigenetic factors as well as cellular and metabolic pathway disturbances in GMD-associated dysfunction of the placenta [12–14]. In addition, impaired glucose uptake and transport in the placenta might also be correlated with GDM. Among the seven isoforms of glucose transporters in the human placenta, glucose transporter 1 (GLUT1), GLUT3, GLUT4, GLUT8, GLUT9, GLUT10, and GLUT12 have a key role in the regulation of glucose transport [15]. Compared to normal pregnancy, GLUT4 [16] and insulin receptor (INSR) were dramatically decreased in the placenta of GDM [12, 17], inhibiting glucose uptake and transport. Upregulation of GLUT1 expression and glucose transfer was also shown in GDM trophoblast cells [18]. However, few studies have revealed placental glucose uptake and transport mechanisms in GDM. Targeting high glucose (HG)-induced trophoblast dysfunction may be an effective strategy for investigating the pathogenesis of GDM.

The mucin (MUC) family includes MUC1-MUC22, and it provides a protective effect for epithelial cells of various organs through secretion and binding mode [19] [20]. As one of the membrane-bound mucins, MUC1 is regarded as a sensor of the external environment and can deliver signals to cells [21]. MUC1 has been found in human trophoblasts, and its expression is increased during placental development. Previous studies have shown that MUC1 could abnormally inhibit trophoblast cell invasion in preeclampsia [22–24]. Meanwhile, MUC1 has been shown to play significant roles in many cellular events, including cell proliferation, apoptosis, adhesion, and invasion [25–27]. However, the action and specific regulatory mechanism of MUC1 in GDM are still not fully elucidated.

β-catenin, as the crucial factor in the Wnt/β-catenin signaling pathway, has a special binding site with MUC1. A previous study demonstrated that MUC1 could stimulate the Wnt/β-catenin pathway and its downstream genes (such as TCF4, CyclinD1, c-Myc, etc.) [28, 29]. In the canonical pathway, Wnt allows β-catenin to dissociate from GSK-3β, leading the stability and accumulation of β-catenin in the cytosol and nucleus [30]. Wnt3a stabilizes β-catenin through the dissociation between β-catenin and glycogen synthase kinase-3β (GSK-3β), which suppresses the phosphorylation and degradation of β-catenin [31]. When Wnt signaling is activated, GSK-3β activity is inhibited, which is mediated through phosphorylation at serine 9, indicating a link between GSK-3β phosphorylation (p-GSK-3β) and the downstream activation of Wnt target genes [32, 33]. We hypothesized that there was a potential correlation between MUC1 levels and the pathology of GDM by activating the Wnt/β-catenin pathway. In this study, HG (30 mM glucose)-induced trophoblast cells (HTR-8/SVneo cells) were used to mimic GDM conditions in vitro. We aimed to investigate the specific role of MUC1 in GDM and to explore the underlying mechanism of MUC1 in placental trophoblast dysfunction during GDM.

## Results

### MUC1 expression was significantly increased in GDM patients, and trophoblast dysfunction was induced in GDM

**Fig. 1:**
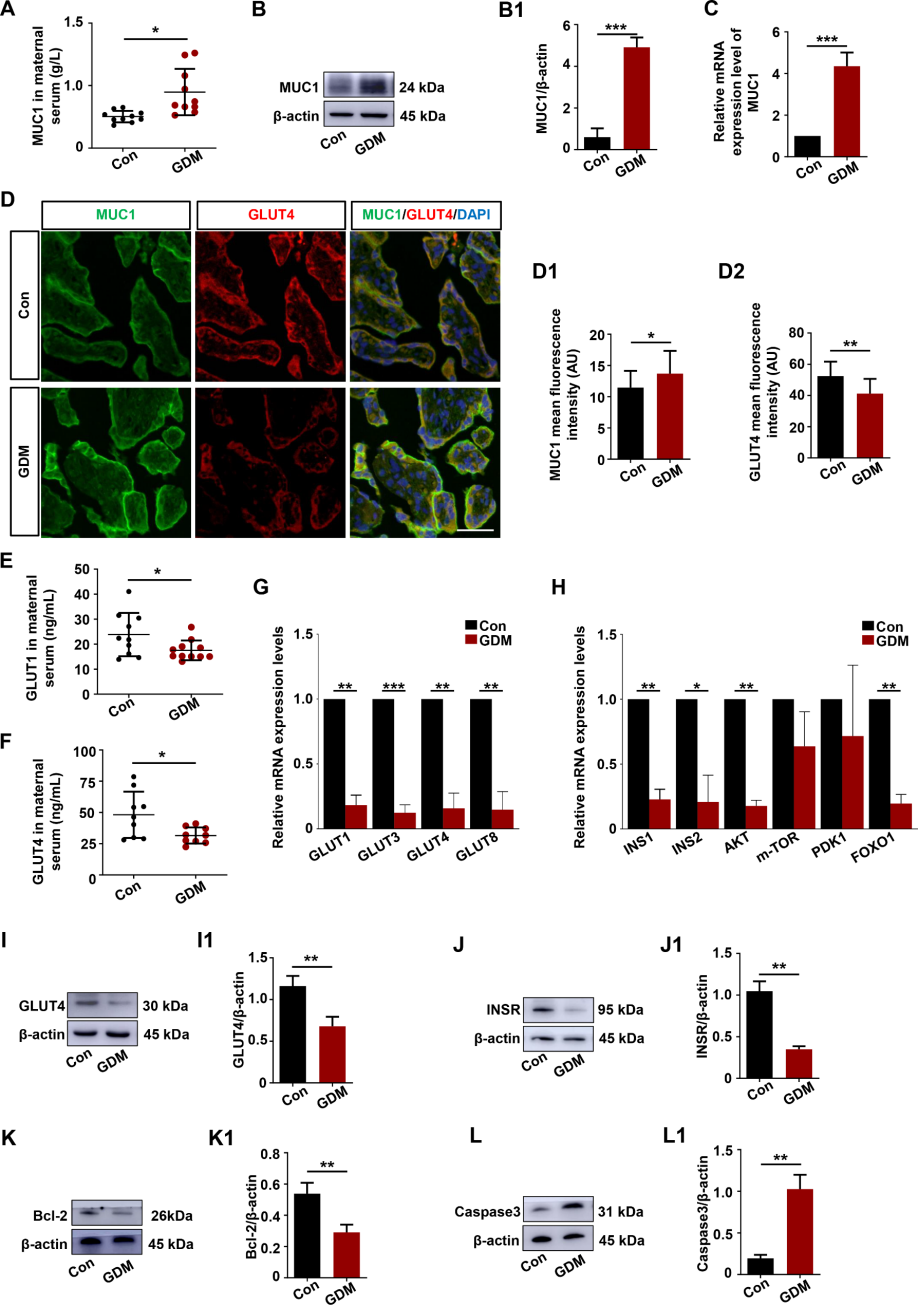
Assessing MUC1 expression levels with glucose uptake- and apoptosis-related proteins in control and GDM placentas. **A**: Determination of the MUC1 level in maternal plasma in the control and GDM groups by ELISA. **B, B1**: Western blotting data showing the expression of MUC1 in the placentas (B), and B1 shows the quantitative analysis of the control and GDM groups. **C**: Quantitative PCR data showing the mRNA expression of MUC1 in control and GDM placentas. **D**: Representative immunofluorescence staining of MUC1 and GLUT4 in the cross-sections of the Control and GDM placentas (counterstained with DAPI). **D1-D2**: Bar charts show the MUC1 and GLUT4 mean fluorescence intensity in Control and GDM placentas. **E-F**: Determination of GLUT1 and GLUT4 levels in maternal plasma in the control and GDM groups by ELISA. **G**: Quantitative PCR data showing the mRNA expression of GLUT1, GLUT3, GLUT4 and GLUT8 in control and GDM placentas. **H**: Quantitative PCR data showing the mRNA expression of INSR, INS1, INS2, AKT, mTOR, PDK1 and FOXO1 in control and GDM placentas. **I-L, I1-L1**: Western blotting data showing the expression of GLUT4 (I), INSR (J), Bcl-2 (K) and Caspase3 (L) in the placentas, and I1-L1 shows the quantitative analysis of the Control and GDM groups, respectively. All statistical data are presented as the mean ± SD. *P < 0.05, **P < 0.01, ***P < 0.001. Scale bar = 250 μm in D

In this study, we demonstrated that the maternal plasma MUC1 levels in the third trimester were significantly elevated in GDM patients (GDM group) compared to healthy subjects (Con group) with ELISA (Fig. 1A). To investigate the expression level of MUC1 in normal and GDM placentas, Western blotting and quantitative PCR (q-PCR) were performed. MUC1 protein and mRNA expression in GDM placentas was higher than that in normal placentas (Fig. 1B-B1, C). Double immunofluorescence staining of MUC1 and GLUT4 showed that there was significantly higher expression of MUC1 and lower expression of GLUT4 in trophoblast cells in GDM placentas than in normal placentas (Fig. 1D, D1-D2). To assess some aspect of glucose uptake, the levels of GLUT1 and GLUT4 in normal and GDM plasma were measured, and the results showed that the levels of both GLUT1 and GLUT4 were decreased in GDM patients, suggesting impaired glucose transport (Fig. 1E-F). Using the q-PCR approach, we demonstrated that the mRNA expression of glucose transporters (e.g., GlUT1, GLUT3, GLUT4, GLUT8) and the key genes of insulin signaling (e.g., INSR, INS1, INS2, AKT, FOXO1) were significantly decreased in GDM placentas (Fig. 1G-H). Meanwhile, the protein expression determined by Western blotting showed that the expression of key glucose uptake genes GLUT4 and INSR was decreased (Fig. 1I-I1, J-J1). In addition, Bcl-2, a marker of resistance to apoptosis, was decreased (Fig. 1K-K1), and Caspase3, an apoptotic marker, was increased (Fig. 1L-L1) in GDM placentas. These data suggested that there was a significant increase in MUC1 expression in both the plasma and placentas in GDM patients, and impaired glucose uptake and apoptosis were induced in GDM placentas.

### Wnt/β-catenin signaling pathway was activeted in GDM

**Fig. 2:**
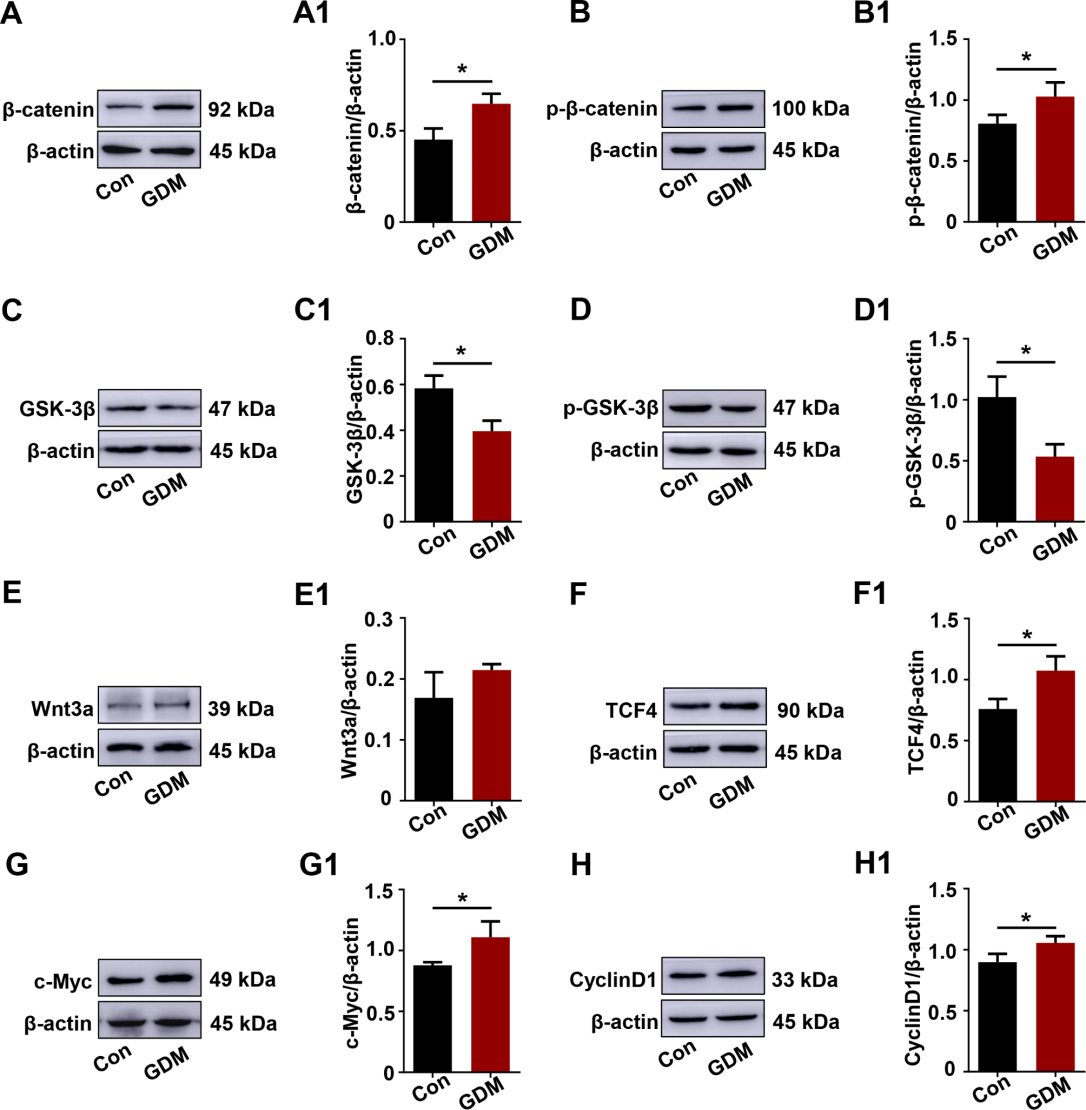
Assessing the Wnt/β-catenin signaling pathway in control and GDM placentas. **A-H, A1-H1**: Western blotting data showing the expression of β-catenin (A), p-β-catenin (B), GSK-3β (C), p-GSK-3β (D), Wnt3a (E), TCF4 (F), c-Myc (G), and CyclinD1 (H) in the placentas. A1-H1 shows the quantitative analysis of the control and GDM groups. All statistical data are presented as the mean ± SD. *P < 0.05, **P < 0.01, ***P < 0.001

To investigate the effect of increased MUC1 levels on the Wnt/β-catenin pathway in GDM, we performed Western blotting to analyze the key genes of the Wnt/β-catenin signaling pathway in placental tissue. The expression levels of β-catenin and p-β-catenin were significantly increased in GDM placentas (Fig. 2A-A1, B-B1). As components of the β-catenin destruction complex, GSK3β and p-GSK3β expression decreased in GDM placentas (Fig. 2C-C1, D-D1). In addition, the Wnt3a expression level showed an increasing trend in GDM placentas (Fig. 2E-E1). Meanwhile, downstream genes of Wnt/β-catenin signaling, such as TCF4, c-Myc, and CyclinD1, were all increased in GDM, as the Western blotting results showed (Fig. 2F-H, F1-H1). The above data certainly suggested that the Wnt/β-catenin signaling pathway was activated in GDM placentas.

### The trophoblast dysfunction induced by high glucose could be ameliorated when the Wnt/β-catenin pathway was inhibited in HTR8/SVneo cells

**Fig. 3:**
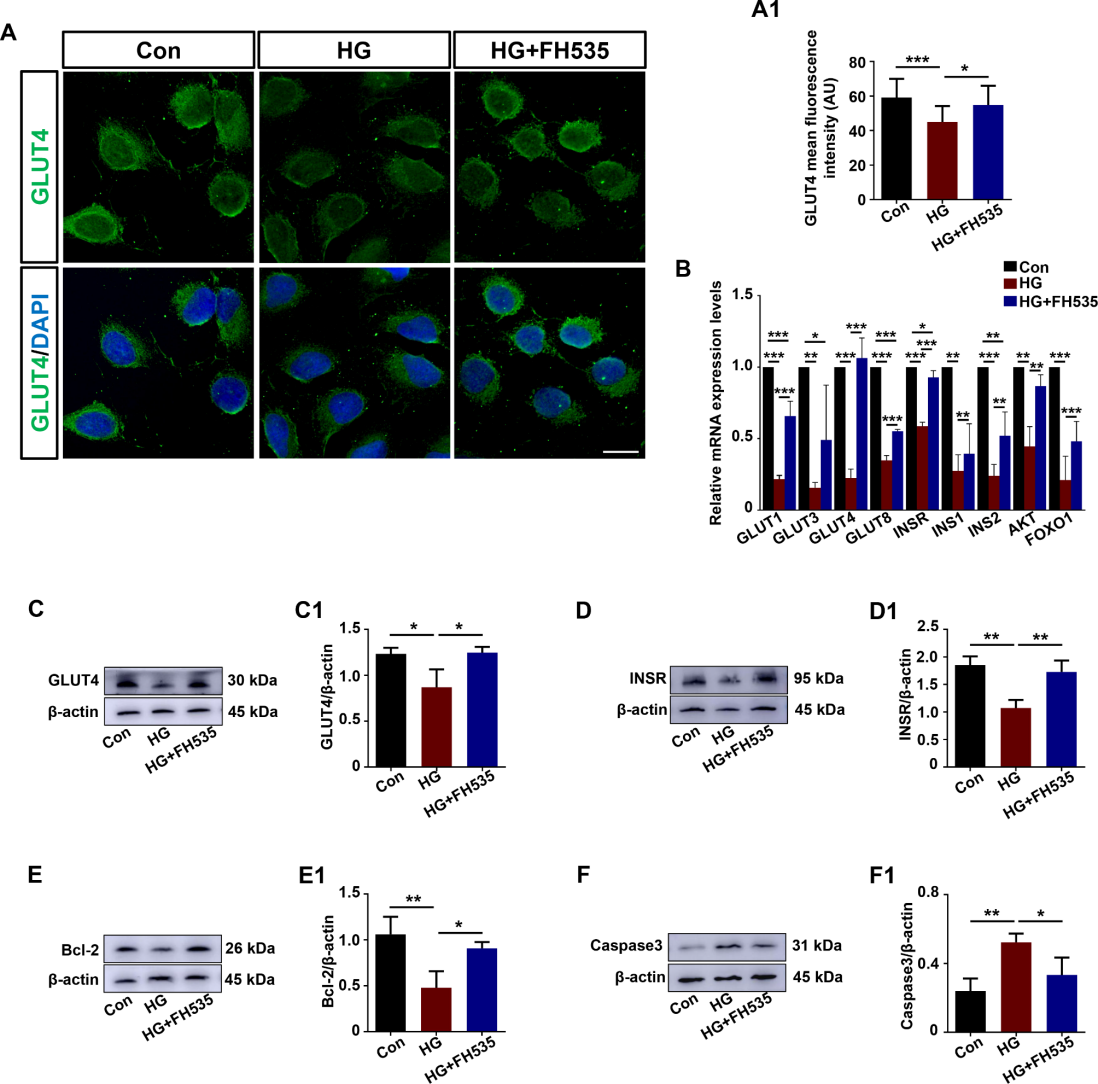
Assessment of glucose uptake- and apoptosis-related proteins in HTR-8/SVneo cells after high glucose and FH535 treatment. **A**: Representative immunofluorescence staining of GLUT4 in HTR-8/SVneo cells of the control, HG and HG + FH535 groups (counterstained with DAPI). **A1**: Bar chart showing the GLUT4 mean fluorescence intensity in HTR-8/SVneo cells of the control, HG and HG + FH535 groups. **B**: Quantitative PCR data showing the mRNA expression of GLUT1, GLUT3, GLUT4, GLUT8, INSR, INS1, INS2, AKT, and FOXO1 in HTR-8/SVneo cells of the control, HG and HG + FH535 groups. **C-F, C1-F1**: Western blotting data showing the expression of GLUT4 (C), INSR (D), Bcl-2 (E), and Caspase3 (F) in HTR-8/SVneo cells, and C1-F1 shows the quantitative analysis of the control, HG and HG + FH535 groups, respectively. All statistical data are presented as the mean ± SD. *P < 0.05, **P < 0.01, ***P < 0.001. HG: high glucose. Scale bar = 20 μm in A

The Wnt/β-catenin pathway was crucial in the maintenance of the physiological functions of trophoblast cells during pregnancy, while the Wnt/β-catenin signaling pathway was activated (Fig. 2) and trophoblast dysfunction was induced in GDM (Fig. 1). We treated HTR8/SVneo cells with HG and FH535 (an inhibitor of the Wnt/β-catenin pathway) in vitro. Immunofluorescence staining of GLUT4 showed that there was significantly lower expression of GLUT4 in trophoblast cells exposed to HG, and with the treatment of HG and FH535, the expression of GLUT4 was reversed (Fig. 3A, A1). To further investigate glucose uptake in trophoblast cells, q-PCR was performed to detect the expression of glucose transporter genes (GLUT1, GLUT3, GLUT4, GLUT8) and insulin signaling-related genes (INSR, INS1, INS2, AKT, FOXO1). The results showed that GLUT1, GLUT3, GLUT4, GLUT8, INSR, INS1, INS2, AKT, and FOXO1 were all decreased in the HG group but efficiently reversed in the HG + FH535 group (Fig. 3B). Impaired glucose uptake and apoptosis were also found in trophoblast cells induced by HG, represented by decreased expression levels of GLUT4, INSR, and Bcl-2 (Fig. 3C-E, C1-E1) and increased expression levels of Caspase-3 (Fig. 3F-F1). With the treatment of FH535, trophoblast dysfunction in a high-glucose environment could be reversed, as the Western blotting data showed (Fig. 3C-F, C1-F1). These results revealed that activation of the Wnt/β-catenin pathway in GDM induced trophoblast dysfunction.

### MUC1 knockdown affected the function of trophoblast cells

**Fig. 4:**
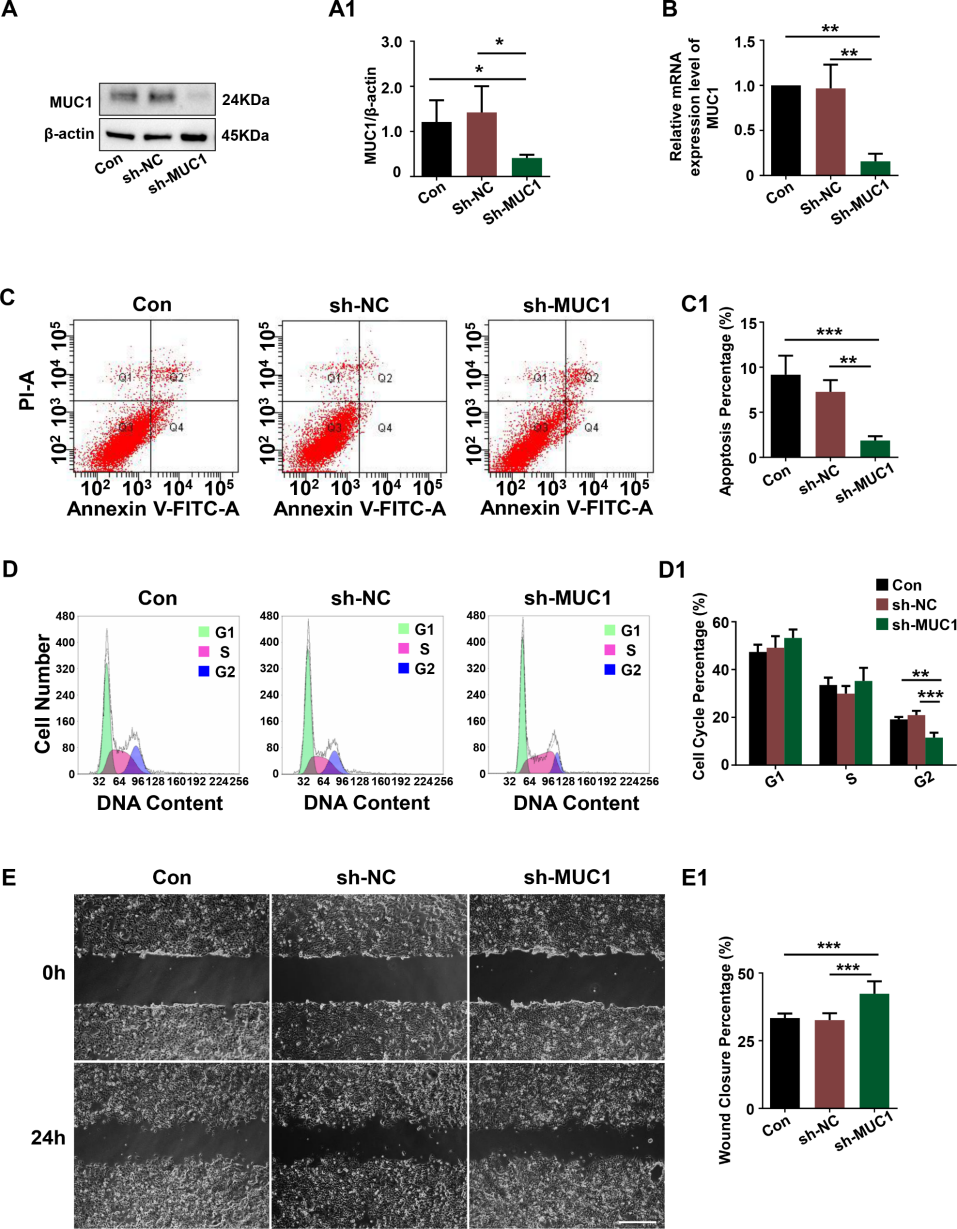
Assessing HTR-8/SVneo cell apoptosis, proliferation and migration after MUC1 knockdown. **A, A1**: Western blotting data showing the expression of MUC1 in HTR-8/SVneo cells, and A1 shows the quantitative analysis of the control, sh-NC and sh-MUC1 groups. **B**: Quantitative PCR data showing the mRNA expression of MUC1 in HTR-8/SVneo cells of the control, sh-NC and sh-MUC1 groups. **C, C1**: Apoptosis of HTR-8/SVneo cells either control cells or transfected with negative control (sh-NC) or sh-MUC1 vectors was determined by flow cytometry using the annexin V-FITC/PI apoptosis assay, and C1 shows the quantitative analysis of the cell apoptosis percentage in the three groups. **D, D1**: Flow cytometry data showing the analysis of DNA contents in HTR-8/SVneo cells either control cells or transfected with negative control or sh-MUC1 vectors, and D1 shows the quantitative analysis of the proportion of cells in each phase of the cell cycle in the three groups. **E, E1**: Representative images of HTR-8/SVneo cells, which were either control cells or negative control (sh-NC) or MUC1 knockdown cells (sh-MUC1), subjected to scratch wounding after 0 and 24 h of incubation. E1 shows the quantitative analysis of the cell mobility percentage in the three groups. All statistical data are presented as the mean ± SD. *P < 0.05, **P < 0.01, ***P < 0.001. Scale bar = 1000 μm in E

Increased expression of MUC1 was observed in trophoblast cells of GDM placentas (Fig. 1B-C), which suggested the role of MUC1 in the regulation of trophoblast cell function. Western blotting analysis showed that the expression level of MUC1 in the sh-MUC1 group was clearly decreased, and there was no significant difference between the control and sh-NC groups, revealing effective MUC1 knockdown in HTR8/SVneo cells (Fig. 4A-A1). Then, we investigated the effect of MUC1 on the proliferation, apoptosis and migration of trophoblast cells by manipulating MUC1 knockdown in HTR8/SVneo cells. Flow cytometry analysis demonstrated that knockdown of MUC1 expression in HTR8/SVneo cells inhibited apoptosis (Fig. 4C-C1) and the cell cycle at the G2 phase (Fig. 4D-D1). In addition, to determine the effects of MUC1 knockdown on trophoblast cell migration, wound healing assays were used, and the results demonstrated that MUC1 knockdown significantly promoted the migratory ability of HTR-8/SVneo cells (Fig. 4E-E1). This evidence suggests that MUC1 is truly associated with trophoblast dysfunction.

### MUC1 knockdown reversed trophoblast cell impairment of glucose uptake and apoptosis promotion induced by high glucose

**Fig. 5:**
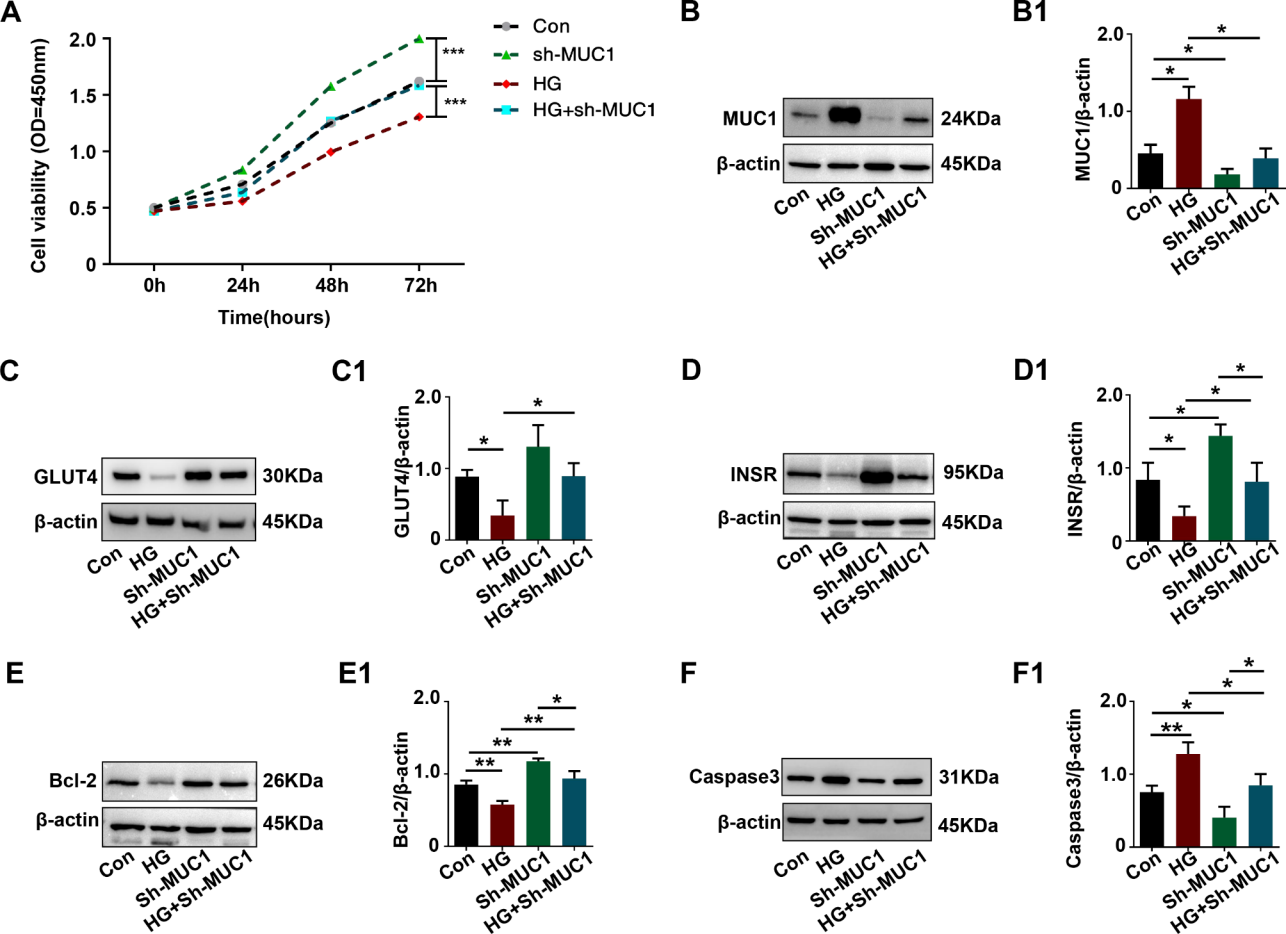
Assessment of glucose uptake- and apoptosis-related proteins in HTR-8/SVneo cells after high glucose treatment and MUC1 knockdown. **A**: Determination of cell viability in HTR-8/SVneo cells from the control, sh-MUC1, HG and HG + sh-MUC1 groups after 0, 24, 48 and 72 h of incubation by CCK-8 assay. **B-F, B1-F1**: Western blotting data showing the expression of MUC1 (B), GLUT4 (C), INSR (D), Bcl-2 (E) and Caspase3 (F) in HTR-8/SVneo cells. B1-F1 shows the quantitative analysis of the control, HG, sh-MUC1 and HG + sh-MUC1 groups. All statistical data are presented as the mean ± SD. *P < 0.05, **P < 0.01, ***P < 0.001. HG: high glucose

To explore the potential role of MUC1 in GDM, high glucose (HG)-induced trophoblast cells (HTR-8/SVneo cells) were used to mimic GDM conditions in vitro. HTR-8/SVneo cells were exposed to 30 mM glucose, and we found that trophoblast cell viability was significantly decreased in the HG group but increased in the MUC1 knockdown group. In addition, MUC1 knockdown reversed the cell viability of trophoblast cells exposed to HG (Fig. 5A). The expression level of MUC1 increased in the HG group but decreased in the sh-MUC1 group, and MUC1 knockdown reversed the increase in MUC1 expression level induced by HG (Fig. 5B-B1). Next, impaired glucose uptake and apoptosis induced by HG were also found in trophoblast cells, represented by decreased GLUT4, INSR, and Bcl-2 and increased Caspase3. In addition, the expression of INSR and Bcl-2 was increased, and the expression of Caspase3 was increased in the sh-MUC1 group compared to the control group. However, MUC1 knockdown reversed the effect induced by HG (Fig. 5C-F, C1-F1). These data showed a similar trend of change in GDM trophoblast dysfunction, which revealed that higher expression of MUC1 in GDM induced impaired glucose uptake and apoptosis of trophoblast cells.

### The Wnt/β-catenin signaling pathway was activated by high glucose and reversed by MUC1 knockdown in HTR8/SVneo cells

**Fig. 6:**
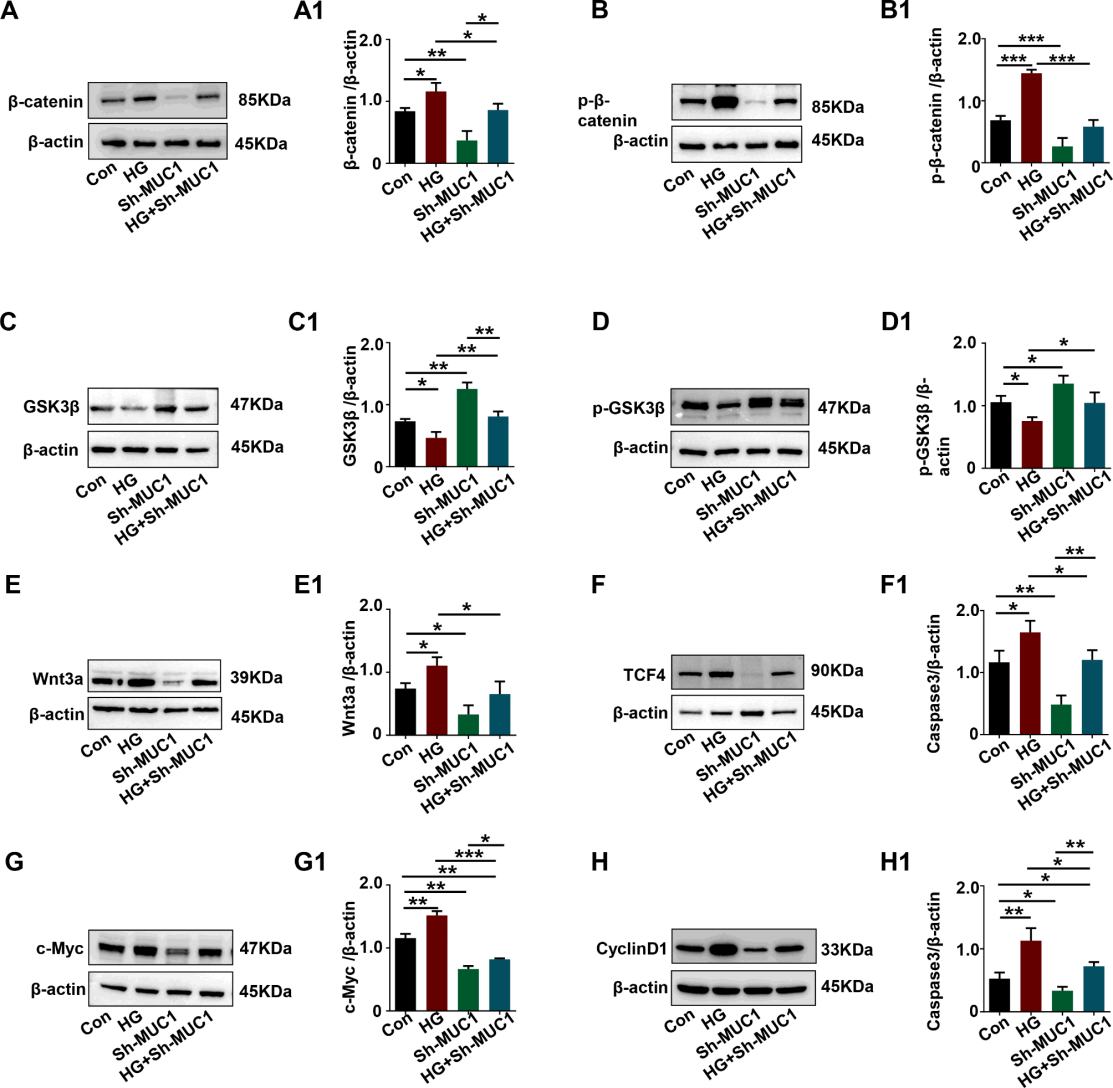
Assessing the Wnt/β-catenin signaling pathway in HTR-8/SVneo cells after high glucose treatment and MUC1 knockdown. **H, A1-H1**: Western blotting data showing the expression of β-catenin (A), p-β-catenin (B), GSK-3β (C), p-GSK-3β (D), Wnt3a (E), TCF4 (F), c-Myc (G), and CyclinD1 (H) in HTR-8/SVneo cells from the control, HG, sh-MUC1 and HG + sh-MUC1 groups, and A1-H1 shows the quantitative analysis of the control, HG, sh-MUC1 and HG + sh-MUC1 groups, respectively. All statistical data are presented as the mean ± SD. *P < 0.05, **P < 0.01, ***P < 0.001. HG: high glucose

To understand the impact of high glucose and MUC1 on Wnt/β-catenin signaling, we compared the expression levels of several members of Wnt/β-catenin signaling by Western blotting. The results revealed that the Wnt/β-catenin pathway was also activated in HG-induced HTR8/SVneo cells, which showed a similar trend to human GDM placentas, while the Wnt/β-catenin pathway was inhibited in MUC1 knockdown HTR8/SVneo cells. The activation and inhibition of Wnt/β-catenin signaling was represented by the corresponding expression levels of β-catenin and p-β-catenin in the HG group and sh-MUC1 group (Fig. 6A-B, A1-B1), as well as the expression levels of GSK3β and p-GSK3β (Fig. 6C-D, C1-D1). Meanwhile, the key gene Wnt3a and downstream genes of Wnt/β-catenin signaling, such as TCF4, c-Myc, and CyclinD1, were all increased in the HG-induced HTR8/SVneo cells but decreased with MUC1 knockdown, as the Western blotting results showed (Fig. 6E-H, E1-H1). However, this effect was completely reversed upon MUC1 knockdown (Fig. 6A-H, A1-H1). MUC1 played important roles in HG-induced HTR8/SVneo cells by regulating Wnt/β-catenin signaling.

## Discussion

GDM is one of the most common metabolism-related diseases during pregnancy and produces substantial amounts of antiangiogenic and proinflammatory factors that impair trophoblast proliferation, migration, and invasion, thereby leading to an unfavorable environment for mental and fetal health [34]. Moreover, the offspring of mothers with GDM are also predisposed to T2D and metabolic syndrome later in life [35]. While the mechanisms of subsequent metabolic disorders in both women with GDM and their offspring are still not clear, impaired placental glucose uptake has increasingly been implicated in this process [36]. Our placental tissue results from women with GDM and trophoblast cell behavior under HG conditions indicate that HG promotes impaired trophoblast cell glucose uptake and promotes apoptosis (Fig. 1). These findings are consistent with those recently reported by Zhang et al. [37].

MUC1 is a highly glycosylated protein that plays different roles in normal and disease states. Overexpression of MUC1 has been found in many types of epithelial cancers, making it a good marker for diagnosis and prognosis in clinical treatment. In tumors, MUC1 is more involved in different signaling pathways that influence and regulate tumor growth, survival, invasion, migration, and apoptosis [38, 39]. It is becoming clear gradually that MUC1 is also highly expressed in GDM, as shown in our data (Fig. 1A-D), but these studies deal only with altered serum levels [40, 41]. However, the mechanism of MUC1 in GDM remains poorly understood. It is known that invasive trophoblasts in macaques express MUC1 and that MUC1 is involved in trophoblast attachment and migration across the endothelium [27]. Trophoblast cells are the most important cells in early pregnancy and are crucial to both placental and fetal development [10, 42]. Elevated expression of MUC1 in the GDM placenta and plasma was found (Fig. 1A-C). More importantly, our results indicated that high MUC1 protein expression in GDM patients might be associated with impaired glucose uptake and apoptosis in placentas. However, MUC1 overexpression suppresses MMP9 activity and reduces the invasion capacity of HTR-8/SVneo cells [23, 43]. In this study, in vitro studies also showed that MUC1 knockdown promoted trophoblast cell proliferation and migration while inhibiting apoptosis (Fig. 4). HG-induced HTR-8/SVneo cell glucose uptake and apoptosis were significantly reversed after knocking down MUC1 expression by shRNA (Fig. 5). Taken together, these results indicate that high expression levels of MUC1 may induce trophoblast dysfunction in GDM.

MUC1 interacts with β-catenin to modulate Wnt signaling [44], directly influencing the transcription of genes associated with cell proliferation, apoptosis, migration, and invasion [45]. Previous evidence indicated that phosphorylation of Ser44 by GSK3β decreases the binding of MUC1 with β-catenin [29]. The Wnt/β-catenin pathway has been shown to play roles in trophoblastic cell differentiation and placental development in pregnant women [46]. Accumulating data indicate that abnormal expression of Wnt/β-catenin signaling might be associated with the pathogenesis of GDM [47]. In the present study, we found that in addition to the increased expression of active β-catenin and p-β-catenin, GSK-3β and p-GSK-3β were also significantly reduced in the placentas of GDM (Fig. 2) and HG-induced trophoblast cells (Fig. 6) compared to those of the control groups. Changes in other genes associated with the Wnt/β-catenin pathway were also observed, including upregulation of Wnt3a, TCF4, c-Myc, and CyclinD1, suggesting regulation of Wnt expression in GDM placentas (Fig. 2) and HG-induced trophoblast cells (Fig. 6). MUC1 was overexpressed in HTR8/SVneo cells exposed to HG, which led to the activation of the Wnt/β-catenin pathway and induced impaired glucose uptake and apoptosis promotion, implying the negative effects of excessive MUC1 in GDM. Furthermore, our data also showed that knockdown of MUC1 expression could significantly suppress trophoblast cell survival and migration in vitro and could significantly inhibit the Wnt/β-catenin pathway. Therefore, we believe that MUC1 plays an important role in trophoblast dysfunction and might be a novel therapeutic target for GDM treatment.

Our in vitro cell experiment results showed that MUC1 knockdown reversed the changes in the Wnt/β-catenin pathway induced by HG (Fig. 6) and had potential protective roles in trophoblast cell glucose uptake and survival (Fig. 5). These results revealed that MUC1 played roles in GDM via the Wnt/β-catenin pathway. However, the detailed upstream mechanism of MUC1 regulation in GDM remains to be further explored.

## Conclusions

**Fig. 7:**
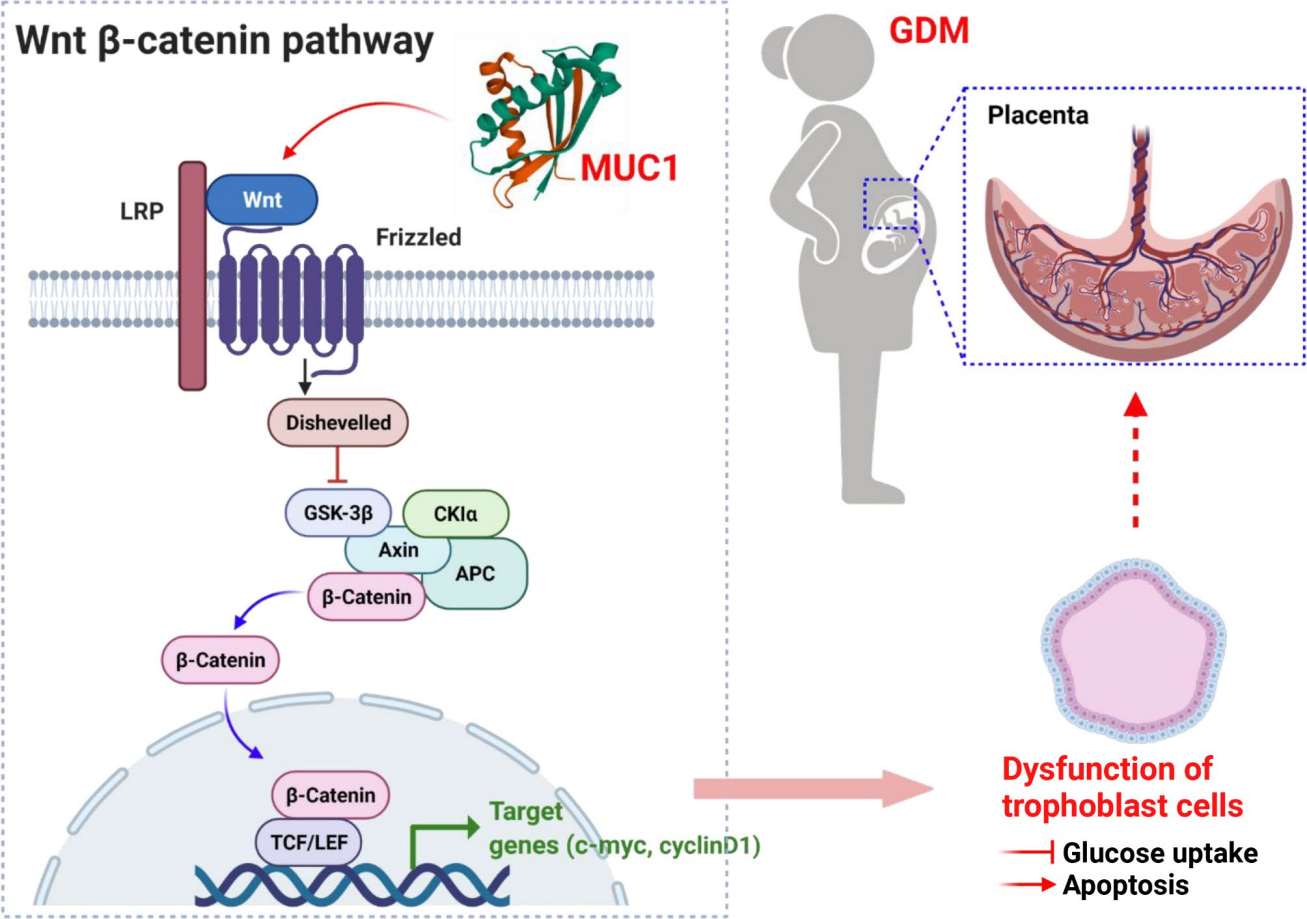
A proposed model that showed the potential mechanisms underlying the role of MUC1 in the pathology of GDM. The proposed mechanism by which MUC1 is involved in the pathology of GDM via the Wnt/β-catenin signaling pathway in trophoblast dysfunction

In summary, (Fig. 7), elevated MUC1 interacts with β-catenin to activate the Wnt/β-catenin pathway, which contributes to overexpression of its target genes c-Myc and CyclinD1 and induces trophoblast cell dysfunction in GDM placentas. Our findings demonstrated for the first time that MUC1 expression increased in placentas and plasma from women with GDM. Moreover, the Wnt/β-catenin pathway was activated in GDM placentas, which showed impaired glucose uptake and apoptosis promotion. In contrast, inhibiting the Wnt/β-catenin pathway by FH535 exerted the opposite effects on HTR-8/SVneo cells induced by HG in vitro. Inhibiting MUC1 expression suppressed the Wnt/β-catenin pathway and reversed trophoblast dysfunction. The results of our investigation suggest an important role of MUC1 in trophoblast dysfunction via the Wnt/β-catenin pathway during pregnancy in GDM.

## Methods

### Human tissue collection

All placenta and plasma samples were obtained from Jan. 2021 to Dec. 2021 at the Department of Gynecology and Obstetrics of the First Affiliated Hospital, Jinan University, China. The GDM diagnostic method was that pregnant women at gestational weeks 24–28 were regularly subjected to a 75-g oral glucose tolerance test, and the diagnosis criteria were according to the American Diabetes Association (ADA) guidelines. To ensure homogeneity between the samples, the GDM pregnancy had no other related diseases, such as preeclampsia or pregestational diabetes. In the normal group, the OGTT assays were normal and without other complications. The clinical characteristics of the pregnant women enrolled in this study are presented in Supplementary file 1: Table S1.

The site of the placenta was located approximately 2 cm from the umbilical insertion site. Blood on the placenta was immediately washed 3 times with phosphate-buffered saline (PBS) and then placed in liquid nitrogen or 4% paraformaldehyde for further studies. In addition, peripheral venous blood was collected from pregnant women in the control group and GDM group on the first day of the last hospital admission (usually within a week) before delivery using anticoagulant tubes, 2 ml peripheral venous blood was collected and then centrifuged (3,000 ×g, 4 °C, 15 min), and then the supernatant (i.e., plasma) was collected and stored at -80 °C for further study.

### Enzyme-linked immunosorbent assay (ELISA)

Whole blood samples were collected from normal and GDM patients. The substances to be tested in the sera were measured by UV spectrophotometry using detection kits according to the manufacturer’s instructions (Mbbiology Biological, Jiangsu, China). The kit details are provided: Human MUC1 ELISA kit (MM-1820H1, MEIMIAN, China), Human GLUT4 ELISA kit (MM-15038H2, MEIMIAN, China), and Human GLUT1 ELISA kit (MM-1186H2, MEIMIAN, China).

### Quantitative PCR (q-PCR) analysis

Total RNA was extracted from human placentas and HTR8 cell lines using a TRIzol kit (Invitrogen, USA). First-strand cDNA was synthesized to a final volume of 20 µl using a SuperScript RIII first-strand kit (Invitrogen, USA). Following reverse transcription, PCR amplification of the cDNA was performed using the corresponding specific primers (note: the sequences of the primers are provided in Supplementary file 1: Table S2). Primers were obtained from Sangon Biotech (Shanghai, China). q-PCR was performed using 2× RealStar Fast SYBR qPCR Mix (A304-10, GenStar, China) in a Bio-Rad S1000TM Thermal cycler (Bio-Rad, USA). The reaction conditions were as follows: 95 °C for 2 min, 40 cycles at 95 °C for 15 s and 60 °C for 30 s, melt curve 65 to 95 °C, increment 0.5 for 5 s. The target gene mRNA expression levels were normalized using 18 S as a reference. The results were analyzed using the 2^−ΔΔCt^ method.

## Immunofluorescence staining

The placental tissues from the normal and GDM groups were fixed in 4% paraformaldehyde, dehydrated, embedded in paraffin wax and serially sectioned at a thickness of 4 μm. Subsequently, the sections were subjected to antigen repair with citric acid, blocked with 10% normal goat serum blocking solution, and incubated with primary antibodies (mouse anti-human MUC1 monoclonal antibody, ab218464, 1:100; rabbit anti-human GLUT4 monoclonal antibody, ab33780, 1 µg/ml) overnight at 4 °C. The sections were stained with fluorescent secondary antibodies (anti-rabbit IgG/FITC or goat anti-mouse IgG/FITC) (Bioss, bs-0295G-FITC, 2 mg/ml). The nuclei were stained with DAPI (Invitrogen). The sections were photographed using a fluorescence microscope (Olympus BX53, Tokyo, Japan). A minimum of 6 random images from 3 samples were analyzed per group.

### Western blotting

Proteins were isolated from the placenta of normal and GDM and HTR-8/SVneo cells using RIPA (Sigma, USA) buffer containing protease and phosphatase inhibitors. Protein concentrations were quantified by BCA assay (Thermo Fisher, A23227, USA). Extracted proteins were separated by 10% SDS‒PAGE and transferred to polyvinylidene difluoride membranes (Millipore, 88,518, USA). The membrane was blocked with 5% nonfat milk, incubated overnight at 4 °C with primary antibody in TBST buffer and shaken gently. After incubation with the secondary antibody, i.e., either horseradish peroxidase goat anti-rabbit IgG (1:3000; Cell Signaling Technology, AB_2099233, USA) or horseradish peroxidase goat anti-mouse IgG (1:3000; Cell Signaling Technology, AB_330924, USA), the samples were treated with SuperSignal™ West Femto chemiluminescent substrate (Thermo Fisher, 34,096, USA) and then photographed by the Gel Doc™ XR + System (Bio-Rad, USA). ImageJ software was used to capture the chemiluminescent signals and analyze the data. The following primary antibodies were used: MUC1 (1:1000, ab109185, Abcam, USA), GLUT4 (1 µg/ml, ab33780, Abcam, USA), INSR (1: 1000, ab227831, Abcam, USA), Bcl-2 (1:1000, ab32124, Abcam, USA), Caspase-3 (1:500, ab13847, Abcam, USA), β-Catenin (1:5000, ab32572, Abcam, USA), p-β-Catenin (1:500, ab75777, Abcam, USA), GSK-3β (1:1000, ab93926, Abcam, USA), p-GSK-3β (1 µg/ml, ab107166, Abcam, USA), Wnt3a (1:1000, ab219412, Abcam, USA), TCF4 (1:10000, ab217668, Abcam, USA), c-Myc (1:1000, ab32072, Abcam, USA), CyclinD1 (1:100, ab16663, Abcam, USA), and β-actin (1:3000, #4970, CST, USA). All experiments were performed with at least three replicates.

### Cell culture and transfection

The human trophoblast cell line HTR8/SVneo was purchased from Zhongqiao Xinzhou Biotechnology Co., Ltd. (Shanghai, China). HTR-8/SVneo cells were cultured in DMEM supplemented with 10% fetal bovine serum (FBS, Gibco) and 1% penicillin‒streptomycin solution (HyClone) at 37°C with 5% CO_2_. HTR-8/SVneo cells were treated for 72 hours with 30 mM D-glucose as the high glucose (HG) group, and normal medium (5.5 mM D-glucose) was used as a control group. HTR-8/SVneo cells (2 × 10^5^ cells/mL) were seeded into 6-well plates and incubated for 24 h until the cells reached 60–70% confluence. Then, the cells were transfected with negative control (sh-NC group) and MUC1-shRNA (sh-MUC1 group) plasmids purchased from Shanghai GeneChem Co., Ltd. (Shanghai, China). The target sequence for MUC1 was as follows: shMUC1: 5’-CCGGCCGGGATACCTACCATCCTATCTCGAGATAGGATGGTAGGTATCCCGGTTTTTG-3’, and the negative control shRNA (sh-NC) sequence was 5’-TTCTCCGAACGTGTCACGT-3’. The vector map and sequence result of the sh-MUC1 plasmid are presented in Supplementary file 1: Results S1-2. The transfection assay was performed according to the manufacturer’s protocol for Lipofectamine 2000. FH535 was purchased from EMD Millipore (Billerica, MA, USA).

### In vitro experiments

#### Flow cytometry (cell apoptosis)

After transfection and treatment, HTR-8/SVneo cells (1 × 10^6^) were collected by centrifugation at 1,000 rpm for 5 min and resuspended in 1 mL of cold PBS. The cells were then stained with an Annexin V-FITC apoptosis kit (88-8005-72, Thermo Fisher, USA) and detected using a FACScan flow cytometer (Becton-Dickinson, San Jose, USA). The acquired data were analyzed using FCS-Express software version 3.0 (De Novo). Three independent experiments were performed.

#### Flow cytometry (cell cycle)

Cell cycle analysis was also detected by flow cytometry. After transfection and treatment, HTR-8/SVneo cells (1 × 10^6^) were collected and washed with cold PBS and then centrifuged, and the supernatant was discarded. Then, 1 ml of -20 °C 70% ethanol was added, gently mixed and fixed overnight at -20 °C. After overnight incubation at -20 °C and washing with PBS, the cells were stained with PI and subjected to flow cytometry. Then, the distribution of cells in the G1, S, and G2/M phases of the cell cycle was determined. Three independent experiments were performed.

*Wound healing assay*: *The* in vitro migration ability of cells was assessed by the wound healing assay. After transfection, 5 × 10^5^ HTR-8/SVneo cells were seeded in 6-well plates and grown to reach confluent monolayers. Then, a 2 µl pipette tip was used to create scratches. Images of migrated cells were recorded at 0–24 h and exported to TIFF with ZEN 2.3 software. The experiments were repeated at least three times. The cell-free area was calculated as the wound area in captured images using ImageJ. The percentage of wound closure was expressed according to the following Eq.  [48]:

$${\text{Wound closure}}\,{\text{\% }}\,{\text{ = }}\,\left( {{{\text{A}}_{0h}} - {{\text{A}}_{24h}}} \right)/{{\text{A}}_{0h}} \times 100\%$$

A_0h_ is the area of the wound measured immediately after scratching (0 h).

A_24h_ was the area of the wound measured 24 h after the scratch was performed.

#### CCK-8 assay

Cell viability was measured using a cell counting kit-8 (CCK8, ab228554, Abcam, UK). Cells from the Con, sh-NC and sh-MUC1 groups were cultured in 96-well plates (2.5 × 10^4^ cells/ml) and treated in the absence or presence of 50 mM D-glucose for 12, 24, 48 and 72 h. Briefly, 10 µl of CCK8 reagent (Dojindo, Kumamoto, Japan) was added to 96-well plates and incubated continually for 2 h at 37 °C. The absorbance values were measured at 450 nm using a Bio-Rad Model 450 microplate reader (Bio-Rad, Hercules, CA, USA).

### Statistical analysis

Construction of statistical charts was performed using the GraphPad Prism 8 software package (GraphPad Software, CA, USA). Statistical analysis was performed using the SPSS 23.0 statistical package program. A comparison of the means between two groups was performed using the Mann‒Whitney U or Student’s t test to estimate the differences. Multiple group comparisons of the means were performed by one-way analysis of variance (ANOVA). Blinded outcome assessment was implemented. All values are presented as the mean ± SD (standard deviation). All statistical data (including n number for the data) are presented in Supplementary file 1: Table S3. P < 0.05 was considered statistically significant.

### Electronic supplementary material

Below is the link to the electronic supplementary material.

Supplementary file 1

## Data Availability

Data sharing is not applicable to this article as no datasets were generated or analysed during the current study.

## List of abbreviations

MUC1: Mucin1
GDM: gestational diabetes mellitus
HG: high glucose
T2DM: type 2 diabetes mellitus
GLUT4: glucose transporter 4
INSR: insulin receptor
GSK-3β: glycogen synthase kinase-3β
PBS: phosphate-buffered saline
