# Supplementary material for: Mucin1 induced trophoblast dysfunction in gestational diabetes mellitus via Wnt/β-catenin pathway
Source: Biol Res. 2023 Aug 22;56:48. doi: 10.1186/s40659-023-00460-3 (PMC10463356; doi:10.1186/s40659-023-00460-3)
Supplement: Supplementary file 1 [file 40659_2023_460_MOESM1_ESM.docx]

**Catalog**

[Supplementary Result 1. The vector map of sh-MUC1 plasmid 2](#_Toc3513)

[Supplementary Result 2. The sequence result of sh-MUC1 plasmid 2](#_Toc28795)

[Table S1. Clinical characteristics of the study population 3](#_Toc1553)

[Table. S2. q-PCR primer sequence 4](#_Toc8241)

[Table. S3. Statistical data (mean ± SD) 5](#_Toc17373)

## Result S1. The vector map of sh-MUC1 plasmid

**
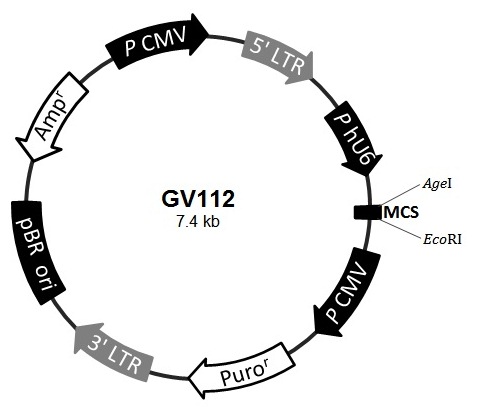
**

## Result S2. The sequence result of sh-MUC1 plasmid

AGGAACTACAACTAAGATTACAAAAACAAATTACAAAAATTCAAAATTTTCGGGTTTATTACAGGGACAGCAGAGATCCAGTTTGGTTAGTACCGGGCCCGCTCTAGACTCGAGCGGCCGCCCCCTTCACCGAGGGCCTATTTCCCATGATTCCTTCATATTTGCATATACGATACAAGGCTGTTAGAGAGATAATTGGAATTAATTTGACTGTAAACACAAAGATATTAGTACAAAATACGTGACGTAGAAAGTAATAATTTCTTGGGTAGTTTGCAGTTTTAAAATTATGTTTTAAAATGGACTATCATATGCTTACCGTAACTTGAAAGTATTTCGATTTCTTGGCTTTATATATCTTGTGGAAAGGACGAAACACCGGCCGGGATACCTACCATCCTATCTCGAGATAGGATGGTAGGTATCCCGGTTTTTGAATTCTCGACCTCGAGACAAATGGCAGTATTCATCCACGAATTCGGATCCATTAGGCGGCCGCGTGGATAACCGTATTACCGCCATGCATTAGTTATTAATAGTAATCAATTACGGGGTCATTAGTTCATAGCCCATATATGGAGTTCCGCGTTACATAACTTACGGTAAATGGCCCGCCTGGCTGACCGCCCAACGACCCCCGCCCATTGACGTCAATAATGACGTATGTTCCCATAGTAACGCCAATAGGGACTTTCCATTGACGTCAATGGGTGGAGTATTTACGGTAAACTGCCCACTTGGCAGTACATCAAGTGTATCATATGCCAAGTACGCCCCCTATTGACGTCAATGACGGTAAATGGCCCGCCTGACATTATGCCCAGTACATGACCTTATGGGACTTTCCTACTTGGCAGTACATCTACGTATTAGTCATCGCTATTACCATGGTGATGCGGTTTTGGCAGTACATCAATGGGCGTGATAGCGGTTTGACTCACGGGGATTTCCAAGTCTCACCCATTGACGTCATGGGAGTTTGTTTGGCACAAATCACGGA

## Table S1. Clinical characteristics of the study population

| **Variables** | **Con**  **n=10** | **GDM**  **n=10** | ***P*-value** |
| --- | --- | --- | --- |
| Gestational weeks at delivery (week) | 40.03 ± 0.41 | 39.54 ± 0.61 | 0.0627 |
| Maternal age (years) | 28.5 ± 4.1 | 30.5 ± 3.14 | 0.2607 |
| Weight (kg) | 60.29 ± 7.65 | 68.22 ± 7.94 | 0.0447* |
| Height (cm) | 160.79 ± 3.23 | 159.55 ± 4.83 | 0.5302 |
| BMI (kg/m^2^) | 23.48 ± 2.89 | 26.75 ± 2.51 | 0.0194* |
| Systolic blood pressure (mmHg) | 115.1 ± 7.63 | 117.1 ± 10.49 | 0.6493 |
| Diastolic blood pressure (mmHg) | 77 ± 10.78 | 73.2 ± 8.66 | 0.4204 |
| MAP (mmHg) | 88.43 ± 19.48 | 86.37 ± 8.24 | 0.6319 |
| OGTT-fasting (mM) | 4.29 ± 0.25 | 4.64 ± 1.11 | 0.37 |
| OGTT-1 hour (mM) | 6.19 ± 1.32 | 10.74 ± 1.5 | <0.001******* |
| OGTT-2 hour (mM) | 6.13 ± 1.09 | 8.61 ± 1.05 | <0.001******* |
| Birthweight (kg) | 3.31 ± 0.39 | 3.24 ± 0.25 | 0.6698 |
| Placental weight (g) | 527 ± 110.37 | 556 ± 33.82 | 0.4608 |

Note: A t-test was used to analyse the normally distributed continuous variables, and the Mann‒Whitney U test was used to analyse the nonnormally distributed data. *p＜0.05，***p＜0.001. Con, Control; GDM, Gestational diabetes mellitus; Weight, the maternal weight in the first day of the latest hospital admission (usually occur within a week before delivery); BMI, body mass index; MAP, mean arterial pressure; OGTT, Oral Glucose Tolerance Test. Values are mean ± S.E.M.

**Table. S2. q-PCR primer sequence**

| Gene | Sapiens |  | Primer |
| --- | --- | --- | --- |
| MUC1 | Human | F | CCTACCATCCTATGAGCGAGTAC |
|  |  | R | GCTGGGTTTGTGTAAGAGAGGC |
| GLUT1 | Human | F | CACTCCTGTTACTTACCTAA |
|  |  | R | CACTTACTTCTGTCTCACT |
| GLUT3 | Human | F | GACCCAGAGATGCTGTAATGGT |
|  |  | R | GGGGTGACCTTCTGTGTCCC |
| GLUT4 | Human | F | CTTCCAACAGATAGGCTCCG |
|  |  | R | CCCCAATGTTGTACCCAAAC |
| GLUT8 | Human | F | TCCTGGTTCGGGGCTGTC |
|  |  | R | GAGCACAGCAAGAGGCTCAG |
| INS1 | Human | F | AGTCTGTCGTCCAGTAGCACCA |
|  |  | R | ACTGGAGCCATACTCATCCGAG |
| INS2 | Human | F | CCTGCCCCCTGCCAACACCT |
|  |  | R | TGTGACATCCTGGTGATAAAGCC |
| AKT2 | Human | F | CATCCTCATGGAAGAGATCCGC |
|  |  | R | GAGGAAGAACCTGTGCTCCATG |
| mTOR | Human | F | AGCATCGGATGCTTAGGAGTGG |
|  |  | R | CAGCCAGTCATCTTTGGAGACC |
| PDK1 | Human | F | CATGTCACGCTGGGTAATGAGG |
|  |  | R | CTCAACACGAGGTCTTGGTGCA |
| FOXO1 | Human | F | CTACGAGTGGATGGTCAAGAGC |
|  |  | R | CCAGTTCCTTCATTCTGCACACG |

# Table. S3. Statistical data (mean ± SD)

| Fig. 1 | Statistical index | | Con | | GDM | | P | |  |
| --- | --- | --- | --- | --- | --- | --- | --- | --- | --- |
| A | MUC1 in maternal serum (g/L) | | 0.50 ± 0.09, N=10 | | 0.90 ± 0.37, N=10 | | 0.009 | |  |
| B1 | MUC1/β-actin | | 0.60 ± 0.42, N=3 | | 4.91 ± 0.47, N=3 | | < 0.001 | |  |
| C | Relative mRNA level of MUC1 | | 1.00 ± 0.00 N=3 | | 4.35 ± 0.65, N=3 | | < 0.001 | |  |
| D1 | MUC1 mean fluorescence intensity (AU) | | 11.45 ± 2.68, N=18 | | 13.68 ± 3.67, N=18 | | 0.045 | |  |
| D2 | GLUT4 mean fluorescence intensity (AU) | | 52.41 ± 9.28 N=18 | | 41.22 ± 9.46 N=18 | | 0.001 | |  |
| E | GLUT1 in maternal serum (ng/mL) | | 23.87 ± 8.67, N=10 | | 17.53 ± 3.96, N=11 | | 0.041 | |  |
| F | GLUT4 in maternal serum (ng/mL) | | 48.08 ± 18.51, N=9 | | 31.55 ± 6.46, N=9 | | 0.03 | |  |
| G | Relative mRNA expression levels | GLUT1 | 1.00 ± 0.00, N=3 | | 0.18 ± 0.08 N=3 | | 0.003 | |  |
|  |  | GLUT3 | 1.00 ± 0.00, N=3 | | 0.12 ± 0.06, N=3 | | < 0.001 | |  |
|  |  | GLUT4 | 1.00 ± 0.00, N=3 | | 0.16 ± 0.12, N=3 | | 0.009 | |  |
|  |  | GLUT8 | 1.00 ± 0.00, N=3 | | 0.15 ± 0.14, N=3 | | 0.009 | |  |
| H | Relative mRNA expression levels | INS1 | 1.00 ± 0.00, N=3 | | 0.23 ± 0.08, N=3 | | 0.003 | |  |
|  |  | INS2 | | 1.00 ± 0.00, N=3 | | 0.21 ± 0.12, N=3 | | < 0.022 | |
|  |  | AKT | 1.00 ± 0.00, N=3 | | 0.18 ± 0.04, N=3 | | 0.001 | |  |
|  |  | mTOR | 1.00 ± 0.00, N=3 | | 0.64 ± 0.27, N=3 | | 0.142 | |  |
|  |  | PDK1 | 1.00 ± 0.00, N=3 | | 0.71 ± 0.55, N=3 | | 0.463 | |  |
|  |  | FOXO1 | 1.00 ± 0.00, N=3 | | 0.20 ± 0.07, N=3 | | 0.003 | |  |
| I | GLUT4/β-actin | | 1.16 ± 0.12, N=3 | | 0.68 ± 0.11, N=3 | | 0.008 | |  |
| J | INSR/β-actin | | 1.05 ± 0.12, N=3 | | 0.35 ± 0.04, N=3 | | 0.001 | |  |
| K | Bcl-2/β-actin | | 0.54 ± 0.07, N=3 | | 0.29 ± 0.05, N=3 | | 0.007 | |  |
| L | Caspase3/β-actin | | 0.19 ± 0.04, N=3 | | 1.03 ± 0.17, N=3 | | 0.001 | |  |

| Fig. 2 | Statistical index | Con | GDM | P |
| --- | --- | --- | --- | --- |
| A1 | β-catanin/β-actin | 0.45 ± 0.06, N=3 | 0.65 ± 0.06, N=3 | 0.014 |
| B1 | p-β-catanin/β-actin | 0.81 ± 0.07, N=3 | 1.02 ± 0.12, N=3 | 0.048 |
| C1 | GSK-3β/β-actin | 0.58 ± 0.06, N=3 | 0.40 ± 0.05, N=3 | 0.011 |
| D1 | p-GSK-3β/β-actin | 1.02 ± 0.17 N=3 | 0.53 ± 0.10 N=3 | 0.013 |
| E1 | Wnt3a/β-actin | 0.17 ± 0.04,  N=3 | 0.21 ± 0.01,  N=3 | 0.142 |
| F1 | TCF4/β-actin | 0.76 ± 0.08, N=3 | 1.10 ± 0.12, N=3 | 0.019 |
| G1 | c-Myc/β-actin | 0.88 ± 0.03, N=3 | 1.11 ± 0.13, N=3 | 0.04 |
| H1 | CyclinD1/β-actin | 0.90 ± 0.07, N=3 | 1.06 ± 0.06, N=3 | 0.036 |

| Fig. 3 | Statistical index | | Con | HG | HG+FH535 | P |
| --- | --- | --- | --- | --- | --- | --- |
| A1 | GLUT4 mean fluorescence intensity (AU) | | 59.15 ± 10.81, N=20 | 45.00 ± 9.25, N=20 | 54.87 ± 11.13, N=20 | Con & HG, P<0.001;  Con & HG+FH535, P=0.402;  HG & HG+FH535 P=0.011. |
| B | Relative mRNA expression levels | GLUT1 | 1.00 ± 0.00, N=3 | 0.22 ± 0.03, N=3 | 0.66 ± 0.10, N=3 | Con & HG, P<0.001;  Con & HG+FH535, P<0.001;  HG & HG+FH535 P< 0.001. |
|  |  | GLUT3 | 1.00 ± 0.00, N=3 | 0.16 ± 0.04, N=3 | 0.49 ± 0.38, N=3 | Con & HG, P=0.003;  Con & HG+FH535, P=0.031;  HG & HG+FH535 P=0.115. |
|  |  | GLUT4 | 1.00 ± 0.00, N=3 | 0.22 ± 0.06, N=3 | 1.06 ± 0.14, N=3 | Con & HG, P<0.001;  Con & HG+FH535, P=0.409;  HG & HG+FH535 P<0.001. |
|  |  | GLUT8 | 1.00 ± 0.00, N=3 | 0.35 ± 0.03, N=3 | 0.55 ± 0.01, N=3 | Con & HG, P<0.001;  Con & HG+FH535, P<0.001;  HG & HG+FH535 P< 0.001. |
|  |  | INSR | 1.00 ± 0.00, N=3 | 0.59 ± 0.03, N=3 | 0.93 ± 0.05, N=3 | Con & HG, P<0.001;  Con & HG+FH535, P=0.034;  HG & HG+FH535 P< 0.001. |
|  |  | INS1 | 1.00 ± 0.00, N=3 | 0.27 ± 0.11, N=3 | 0.39 ± 0.21, N=3 | Con & HG, P=0.001;  Con & HG+FH535, P=0.323;  HG & HG+FH535 P=0.002. |
|  |  | INS2 | 1.00 ± 0.00, N=3 | 0.24 ± 0.08, N=3 | 0.52 ± 0.18, N=3 | Con & HG, P<0.001;  Con & HG+FH535, P=0.001;  HG & HG+FH535 P=0.018. |
|  |  | AKT | 1.00 ± 0.00, N=3 | 0.45 ± 0.14, N=3 | 0.87 ± 0.08, N=3 | Con & HG, P<0.001;  Con & HG+FH535, P=0.128;  HG & HG+FH535 P=0.001. |
|  |  | FOXO1 | 1.00 ± 0.00, N=3 | 0.21 ± 0.17, N=3 | 0.87 ± 0.08, N=3 | Con & HG, P<0.001;  Con & HG+FH535, P=0.182;  HG & HG+FH535 P<0.001. |
| C1 | GLUT4/β-actin | | 1.23 ± 0.07, N=3 | 0.87 ± 0.20, N=3 | 1.25 ± 0.06, N=3 | Con & HG, P =0.012;  Con & HG+FH535, P=0.899;  HG & HG+FH535 P=0.010. |
| D1 | INSR/β-actin | | 1.85 ± 0.16, N=3 | 1.07 ± 0.15, N=3 | 1.73 ± 0.20, N=3 | Con & HG, P =0.001;  Con & HG+FH535, P=0.406;  HG & HG+FH535 P=0.003. |
| E1 | Bcl-2/β-actin | | 1.06 ± 0.19, N=3 | 0.48 ± 0.18, N=3 | 0.91 ± 0.07, N=3 | Con & HG, P =0.004;  Con & HG+FH535, P=0.280;  HG & HG+FH535 P=0.015. |
| F1 | Caspase3/β-actin | | 0.24 ± 0.07, N=3 | 0.52 ± 0.05, N=3 | 0.33 ± 0.10, N=3 | Con & HG, P =0.004;  Con & HG+FH535, P=0.193;  HG & HG+FH535 P=0.024. |

| Fig. 4 | Statistical index | | Con | sh-NC | sh-MUC1 | P |
| --- | --- | --- | --- | --- | --- | --- |
| A1 | MUC1/β-actin | | 1.21 ± 0.48,  N=3 | 1.42 ± 0.58,  N=3 | 0.37 ± 0.12,  N=3 | Con&sh-NC, P =0.65;  Sh-NC&sh-MUC1, P =0.04;  Con&sh-MUC1, P=0.047. |
| B | Relative mRNA expression level of MUC1 | | 1.00 ± 0.00, N=3 | 0.97 ± 0.26, N=3 | 0.16 ± 0.08, N=3 | Con&sh-NC, P =0.807;  Sh-NC&sh-MUC1, P =0.001;  Con&sh-MUC1, P=0.001. |
| C1 | Apoptosis Percentage (%) | | 9.17 ± 2.11, N=3 | 7.27 ± 1.31, N=3 | 0.16 ± 0.08, N=3 | Con&sh-NC, P =0.156;  Sh-NC&sh-MUC1, P =0.01;  Con&sh-MUC1, P<0.001. |
| D1 | Cell Cycle Percentage (%) | G1 | 47.37 ± 3.07, N=3 | 49.13 ± 4.82, N=3 | 53.23 ± 3.56, N=3 | Con&sh-NC, P =0.598;  Sh-NC&sh-MUC1, P =0.244;  Con&sh-MUC1, P=0.114. |
|  |  | S | 33.5 ± 3.12, N=3 | 29.93 ± 3.19, N=3 | 35.23 ± 5.51, N=3 | Con&sh-NC, P =0.327;  Sh-NC&sh-MUC1, P =0.164;  Con&sh-MUC1, P=0.623. |
|  |  | G2 | 19.13 ± 1.05, N=3 | 20.93 ± 1.78, N=3 | 11.53 ± 2.06, N=3 | Con&sh-NC, P =0.238;  Sh-NC&sh-MUC1, P<0.001;  Con&sh-MUC1, P=0.001. |
| E1 | Wound Closure Percentage (%) | | 33.40 ± 1.67, N=12 | 32.62 ± 2.52, N=12 | 42.34 ± 4.61, N=12 | Con&sh-NC, P =0.555;  Sh-NC&sh-MUC1, P<0.001;  Con&sh-MUC1, P=P<0.001. |

| Fig. 5 | Statistical index | | Con | HG | sh-MUC1 | HG+MUC1 | P |
| --- | --- | --- | --- | --- | --- | --- | --- |
| A | Cell viability （OD=450nm） | 0 h | 0.50 ± 0.00, N=3 | 0.47 ± 0.01, N=3 | 0.50 ± 0.01, N=3 | 0.47 ± 0.02, N=3 | Con&HG, P =0.008;  Con&sh-MUC1, P =0.699;  Con&HG+sh-MUC1, P=0.008;  HG&HG+sh-MUC1, P=0.015;  Sh-MUC1&HG+sh-MUC1, P=0.015. |
|  |  | 24 h | 0.71 ± 0.01, N=3 | 0.56 ± 0.02, N=3 | 0.84 ± 0.02, N=3 | 0.64 ± 0.02, N=3 | Con&HG, P<0.001;  Con&sh-MUC1, P<0.001;  Con&HG+sh-MUC1, P=0.001;  HG&HG+sh-MUC1, P=0.001;  Sh-MUC1&HG+sh-MUC1, P<0.001. |
|  |  | 48 h | 1.25 ± 0.00, N=3 | 0.99 ± 0.02, N=3 | 1.58 ± 0.02, N=3 | 1.28 ± 0.03, N=3 | Con&HG, P<0.001;  Con&sh-MUC1, P<0.001;  Con&HG+sh-MUC1, P=0.315;  HG&HG+sh-MUC1, P<0.001;  Sh-MUC1&HG+sh-MUC1, P<0.001. |
|  |  | 72 h | 1.62 ± 0.00, N=3 | 1.31 ± 0.02, N=3 | 2.00 ± 0.02, N=3 | 1.58 ± 0.01, N=3 | Con&HG, P<0.001;  Con&sh-MUC1, P<0.001;  Con&HG+sh-MUC1, P=0.012;  HG&HG+sh-MUC1, P<0.001;  Sh-MUC1&HG+sh-MUC1, P<0.001. |
| B1 | MUC1/β-actin | | 0.45 ± 0.11,  N=3 | 1.16 ± 0.16,  N=3 | 0.18 ± 0.07,  N=3 | 0.39 ± 0.13,  N=3 | Con&HG, P=0.03;  Con&sh-MUC1, P=0.02;  Con&HG+sh-MUC1, P=0.55;  HG&HG+sh-MUC1, P=0.03;  Sh-MUC1&HG+sh-MUC1, P=0.068. |
| C1 | GLUT4/β-actin | | 0.89 ± 0.09,  N=3 | 0.34 ± 0.21, N=3 | 1.30 ± 0.30,  N=3 | 0.89 ± 0.18, N=3 | Con&HG, P =0.015;  Con&sh-MUC1, P =0.08;  Con&HG+sh-MUC1, P=0.97;  HG&HG+sh-MUC1, P=0.27;  Sh-MUC1&HG+sh-MUC1, P=0.11. |
| D1 | INSR/β-actin | | 0.84 ± 0.23,  N=3 | 0.34 ± 0.13, N=3 | 1.44 ± 0.16,  N=3 | 0.81 ± 0.26, N=3 | Con&HG, P =0.033;  Con&sh-MUC1, P =0.021;  Con&HG+sh-MUC1, P=0.911;  HG&HG+sh-MUC1, P=0.48;  Sh-MUC1&HG+sh-MUC1, P=0.23. |
| E1 | Bcl-2/β-actin | | 0.85 ± 0.05,  N=3 | 0.58 ± 0.05, N=3 | 1.17 ± 0.04,  N=3 | 0.93 ± 0.10, N=3 | Con&HG, P =0.003;  Con&sh-MUC1, P =0.001;  Con&HG+sh-MUC1, P=0.272;  HG&HG+sh-MUC1, P=0.005;  Sh-MUC1&HG+sh-MUC1, P=0.02. |
| F1 | Caspase3/β-actin | | 0.76 ± 0.09,  N=3 | 1.28 ± 0.16, N=3 | 0.41 ± 0.15, N=3 | 0.85 ± 0.15, N=3 | Con&HG, P =0.008;  Con&sh-MUC1, P =0.026;  Con&HG+sh-MUC1, P=0.408;  HG&HG+sh-MUC1, P=0.03;  Sh-MUC1&HG+sh-MUC1, P=0.023. |

| Fig. 6 | | Statistical index | | Con | | HG | | sh-MUC1 | | HG+MUC1 | | P |
| --- | --- | --- | --- | --- | --- | --- | --- | --- | --- | --- | --- | --- |
| A1 | | β-catenin/β-actin | | 0.84 ± 0.05,  N=3 | | 1.16 ± 0.14, N=3 | | 0.37 ± 0.15, N=3 | | 0.86 ± 0.10, N=3 | | Con&HG, P =0.02;  Con&sh-MUC1, P =0.007;  Con&HG+sh-MUC1, P=0.771;  HG&HG+sh-MUC1, P=0.04;  Sh-MUC1&HG+sh-MUC1, P=0.01. |
| B1 | p-β-catenin/β-actin | | 0.68 ± 0.07,  N=3 | | 1.44 ± 0.06, N=3 | | 0.26 ± 0.14, N=3 | | 0.58 ± 0.11, N=3 | | Con&HG, P < 0.001;  Con&sh-MUC1, P =0.009;  Con&HG+sh-MUC1, P=0.245;  HG&HG+sh-MUC1, P < 0.001;  Sh-MUC1&HG+sh-MUC1, P=0.33. | |
| C1 | GSK3β/β-actin | | 0.73 ± 0.04,  N=3 | | 0.47 ± 0.10, N=3 | | 1.25 ± 0.10, N=3 | | 0.81 ± 0.08, N=3 | | Con&HG, P =0.0.01;  Con&sh-MUC1, P =0.001;  Con&HG+sh-MUC1, P=0.211;  HG&HG+sh-MUC1, P=0.009;  Sh-MUC1&HG+sh-MUC1, P=0.004. | |
| D1 | p-GSK3β/β-actin | | 1.06 ± 0.10,  N=3 | | 0.76 ± 0.06, N=3 | | 1.35 ± 0.13,  N=3 | | 1.05 ± 0.17, N=3 | | Con&HG, P =0.012;  Con&sh-MUC1, P =0.037;  Con&HG+sh-MUC1, P=0.94;  HG&HG+sh-MUC1, P=0.046;  Sh-MUC1&HG+sh-MUC1, P=0.063. | |
| E1 | Wnt3a/β-actin | | 0.74 ± 0.09,  N=3 | | 1.11 ± 0.13, N=3 | | 0.33 ± 0.15,  N=3 | | 0.65 ± 0.20, N=3 | | Con&HG, P =0.016;  Con&sh-MUC1, P =0.013;  Con&HG+sh-MUC1, P=0.522;  HG&HG+sh-MUC1, P=0.031;  Sh-MUC1&HG+sh-MUC1, P=0.086. | |
| F1 | TCF4/β-actin | | 1.17 ± 0.19,  N=3 | | 1.65 ± 0.19, N=3 | | 0.48 ± 0.15, N=3 | | 1.20 ± 0.16,  N=3 | | Con&HG, P =0.034;  Con&sh-MUC1, P =0.008;  Con&HG+sh-MUC1, P=0.798;  HG&HG+sh-MUC1, P=0.034;  Sh-MUC1&HG+sh-MUC1, P=0.004. | |
| G1 | c-Myc/β-actin | | 1.16 ± 0.07,  N=3 | | 1.52 ± 0.07, N=3 | | 0.66 ± 0.05, N=3 | | 0.82 ± 0.16,  N=3 | | Con&HG, P =0.003;  Con&sh-MUC1, P =0.001;  Con&HG+sh-MUC1, P=0.001;  HG&HG+sh-MUC1, P<0.001;  Sh-MUC1&HG+sh-MUC1, P=0.024. | |
| H1 | CyclinD1/β-actin | | 0.53 ± 0.10,  N=3 | | 1.13 ± 0.20, N=3 | | 0.33 ± 0.06, N=3 | | 0.72 ± 0.07,  N=3 | | Con&HG, P =0.009;  Con&sh-MUC1, P =0.045;  Con&HG+sh-MUC1, P=0.045;  HG&HG+sh-MUC1, P=0.027;  Sh-MUC1&HG+sh-MUC1, P=0.002. | |
